# Does gastric tonometry-guided therapy reduce total mortality in critically ill patients?

**DOI:** 10.1186/s13054-015-0893-x

**Published:** 2015-04-02

**Authors:** Michael G Mythen

**Affiliations:** The Institute of Sport Exercise and Health, University College London, 170 Tottenham Court Road, London, W1T 7HA UK

## Abstract

Zhang and colleagues have recently published a systematic review and meta-analysis of six studies and conclude that ‘gastric tonometry guided therapy can reduce total mortality in critically ill patients’. So why did gastric tonometry come and go, and what can we learn from this piece of modern history?

Gastric tonometry measures the balance between alveolar ventilation, gastric blood flow, and metabolism [1,2]. In the 1990s, gastric tonometry was a fashionable clinical monitor and was incorporated into numerous laboratory and clinical trials [3-6]. Then, soon after a small randomised controlled trial (RCT) of just over 200 patients reported no impact on ICU mortality when gastric tonometry was used to guide therapy, it seemed to disappear as a clinical tool [7]. However, Zhang and colleagues have recently published a systematic review and meta-analysis of these six studies and conclude that ‘gastric tonometry guided therapy can reduce total mortality in critically ill patients’ [1]. So why did gastric tonometry come and go, and what can we learn from this piece of modern history?

Hollow viscus tonometry is a long-established technique. Lavaging a hollow viscus such as the gall bladder or gastrointestinal tract allows an estimate of the partial pressure of gas tension in the wall of the viscus by analysis of the lavage. It was deployed in the stomach over decades, evolving from sampling gastric juice to the use of condoms attached to nasogatric tubes and eventually bespoke modified nasogatric tubes that incorporated a silicone balloon and sampling line. Manual saline tonometry required the balloon to be filled with 2.5 mL saline and, following a dwell time of up to 90 minutes, sampling and analysis using a blood-gas analyser [3-6]. Attention was initially focused on the calculation of ‘gastric intra-mucosal pH’ (or ‘pHi’) by using the gut lumen carbon dioxide (CO_2_) measured by tonometry and the calculated arterial bicarbonate concentration from an arterial sample drawn at the same time. The theory was that, during periods of reduced gastric blood flow, a critical level would be reached below which anaerobic metabolism would be the dominant metabolic pathway for the generation of energy. Anaerobic metabolism generates lactic acid and causes the accumulation of CO_2_.

The first bespoke gastric tonometer was probably launched prematurely as a number of technical glitches, such as the impact of poor sampling technique and temperature on CO_2_ tension, needed to be resolved post-launch. Despite these glitches, ‘pHi’ measurement became popular in clinical observational studies and was demonstrated in major surgery, trauma, and the ICU to be a highly sensitive but less specific predictor of a poor outcome [3-6]. Doubt was cast on the utility of ‘pHi’ as it incorporated both global acid–base balance and regional partial pressure of CO_2_ (PCO_2_) [1,8]. Thus, a metabolic acidosis without an excess accumulation of gastric CO_2_ could result in a low ‘pHi’ that was simply a repackaging of base excess [2,8]. Finally, automated air tonometry was launched [9]. The bespoke tonometer tube was unchanged but now air rather than saline was used to fill the balloon. This facilitated quicker full equilibration and automated sampling and measurement by using a modified end-tidal CO_2_ infra-red analyser [9]. The calculation of ‘pHi’ was abandoned and interest turned to the rise in gastric partial pressure of CO_2_ compared with either the arterial partial pressure of CO_2_ or end-tidal partial pressure of CO_2_, referred to as the PCO_2_ ‘gap’ or ‘gradient’. This again proved to be highly predictive of a poor outcome, particularly in major surgery [9]. So now, at last, we thought we had a user-friendly, automated, robust surrogate measure of ‘end-organ perfusion’ and a growing understanding of the technique and the separation between global haemodynamic variables and splanchnic blood flow. It was demonstrated, for example, that haemorrhage in adult volunteers could be detected by gastric tonometry when commonly measured haemodynamic variables remained unchanged [10] and that if critically ill patients had an abnormal PCO_2_ ‘gap’ they failed to produce gastric acid following pentagastrin stimulation [11]. Furthermore, gut-directed therapy could maintain or correct PCO_2_ ‘gap’ [4,12]. So where did it all go wrong?

I think there were a number of factors. Gastric tonometry was made commercially available before all of the methodological issues had been resolved and this resulted in negative press. Furthermore, evidence-based medicine and the demand for ‘proof’ of safety and efficacy from large RCTs were just emerging. How one should apply these standards to monitors of physiological variables was not and has probably still not been completely resolved. Where should the burden of proof lie? With manufacturers or the clinical community? What would be the cost implications of demanding the equivalent of phase III level of evidence for monitors? Gastric tonometry was caught up in this emerging debate and came off second best. Perhaps the burden lies with the clinical trials, although noble efforts in their day would now be regarded as inadequately designed to answer the question ‘does gastric tonometry guided resuscitation improve ICU survival?’ [1]. The largest of the six studies randomly assigned just 260 patients—some 10- to 20-fold fewer than the numbers one might expect to have to recruit today to answer the same question [1,4]. The recent meta-analysis by Zhang and colleagues concludes (among other things) that ‘in critical care patients, gastric tonometry guided therapy can reduce total mortality’ [1]. On reviewing the results, one can see that six small RCTs were conducted on a diverse range of populations (surgery, trauma, and the ICU). All of the trials were grossly underpowered to determine a possible impact on mortality. However, the point estimates for impact on mortality (Figure three [1]) all favour the intervention, but the confidence intervals are large and cross the line of unity.

I suggest that if we were starting from this point today, we would conclude that there is equipoise, significant uncertainty, and enough evidence to justify asking the question ‘does gastric tonometry-guided therapy reduce total mortality in critically ill patients?’ This question could be answered by a pragmatic, high-quality RCT with patient-centred outcomes, but I doubt it will be.

## Abbreviations

CO_2_: Carbon dioxide
PCO_2_: Partial pressure of carbon dioxide
pHi: Gastric intra-mucosal pH
RCT: Randomised controlled trial.
